# Prevalence of Psychiatric Disorders and Related Factors in Male Prisoners

**DOI:** 10.5812/ircmj.15205

**Published:** 2014-01-05

**Authors:** Zahra Sepehrmanesh, Afshin Ahmadvand, Goudarz Akasheh, Rezvan Saei

**Affiliations:** 1 Department of Psychiatric, Kashan University of Medical Sciences, Kashan, IR Iran

**Keywords:** Prevalence, Mental Disorders, Prisoners

## Abstract

**Background:**

Prisoners are at risk of mental disorders. Therefore attention to mental health of prisoners is important.

**Objectives:**

This study aimed determine to the prevalence of mental disorders among Kashan prisoners.

**Patients and Methods:**

This cross sectional study was carried out in Kashan prison (Iran). 180 Subjects were selected by using stratified random sampling and evaluated with Symptoms Check List-90-Revised (SCL-90-R) questionnaire and clinical interview based on Diagnostic Statistical Manual of Disease-IV (DSM-IV) check list by two psychiatrists. Data were analyzed by SPSS-16 software and Chi square, Kolmogrov Smirnov, Mann-whiteny and Leven tests.

**Results:**

The mean age of prisoners was 31.9 ± 8.96. The prevalence of psychiatric disorders in prisoners was 43.4 %. The most frequent disorders were major depressive disorders (27.9 %), Post traumatic Stress Disorder (17.4%) and substance use disorder (17.4 %). 28.3% of prisoners had personality disorders, that the most prevalent were antisocial personality and borderline personality. The comorbidity of psychiatric disorders was (36 %) in axis I. Suicidal thoughts there were in 44.6 % of prisoners. History of head trauma in Prisoners with psychiatric disorders was (52.2 %). There was significant difference between head trauma and psychiatric disorders (P = 0.05). Significant difference was between marital status and duration of imprisonment with psychiatric disorders P < 0.05. There was not significant difference between type of crime and educational level with psychiatric disorders.

**Conclusions:**

About half of all prisoners suffered from psychiatric disorders; therefore treatment psychiatric disorder in this group is essential for prevention of crime. Prisoners are at risk of mental disorders. Therefore attention to mental health of prisoners is important.

## 1. Background

Mental disorders are one of the most common disorders in the world and prisoner's mental health is a major concern of public health in today society (1). Numerous studies have shown that mental disorders can be found significantly more frequently in prisoners in comparison to normal populations (2). Moore (2013) reported the prevalence of mental disorders is 17% among prisoners (3). Prisoners do not seek help for their psychiatric problems and therefore their needs less assessed and so suicidal behavior in them is common (1, 4). The World statistics indicate that more than 9 million people live on prisons of the world (1). According to the reports; more than 163000 prisons are in Iran (2). This issue that prison populations have less mental health than the general population has been identified in the research of Brinded (2004) (4). Nilsson and Misrachi in their study found that 5.1 % of prisoners had psychosis disorders (5). Brugha (2002) reported the prevalence of mental disorders in prisoners is 10 times more than their staff (6). The prevalence of mental disorders is %66 in prisoners of England and 85 % in Finland (7). The prevalence of mental disorders in Iran's prisons has been reported; 75 % in Shiraz, 88% in Tehran (8, 9). The majority of studies represent that personality disorders and substance abuse disorders are the most frequent disorders in prisoner populations (10-12). The prevalence of personality disorders in Sanandaj prison was reported 88 % (13). These Studies demonstrate that a many prisoners experience mental health problems.

## 2. Objectives

As mental disorders have an essential role in the incidence of crime and since no study has been conducted about prevalence of mental disorder and some risk factors in Kashan (Iran) this research was designed. This study evaluates mental disorders in men prisoners including prevalence mental disorders in axis I and personality disorders in axis II and some risk factors of psychiatric disorders.

## 3. Materials and Methodes

### 3.1. Subjects and Sampling Method

This cross sectional study was conducted on men prisoners in Kashan city /Iran (2011-2012). The sample number (n = 180) was determined with (z = 1.96, q = 0.25, d = 0.05, P = 0.75). The subjects were selected by stratified randomized sampling among prison's wards (N = 535). Inclusion and exclusion criteria were male sex, duration of imprisonment at least 3 months, lack of mental retardation, lack of delirium, dementia and physical disability. After taking formal consent from administrator of prison, the list of prisoners was provided by clinical psychologist of Kashan prison. The proportion of each ward was determined via *ni = ki / N × n*. The participants filled questionnaires after written consent. The information was kept secret. Prisoners who their scores were above cut off point in the SCL-90 test, were referred for clinical interview by two psychiatrists based on DSM-IV check list. Based on SCL-90 questionnaire results, 131 subjects based on inclusion criteria were referred to the psychiatrist for evaluation of mental disorders based on DSM-IV checklist. 21 cases were excluded because of freedom and other problems, therefore 159 subjects were interviewed by two psychiatrists.

### 3.2. Tools

#### 3.2.1. Symptom Checklist 90 Revised (SCL-90-R)

Psychopathological features were assessed with the symptom checklist 90 revised , a self- administered questionnaire used to evaluate the symptoms of psychopathology experienced by individuals even beyond clinically relevant mental disorders. The questionnaire is appropriate for use in both normal and distressed individuals. The questionnaire consist of 90 items concerning an individual's symptom distress. Each item is related on a five-point Iikert scale (0-4). The nine subscales that can be derived from the SCL-90 are:

Somatization (som, 12 items), obsessive compulsive (OBS, 10 items), interpersonal sensitivity (SENS, 9 items), depression (DEP, 13 items), anxiety (ANX, 10 items), anger hostility (HOS, 6 items), phobic anxiety (PHOB, 7 items), Paranoid ideation (PAR, 6 items) and psychotic (PSYC, 10 items). The final score of the GSI, which represents the average severity score of all the 90 items of the questionnaire, is thought to be a reliable measure of psychological distress. The cut off point for GSI used in this study is 0.7, as indicated by the existing literature. (14, 15). Reliability of this scale has been reported 0.90 in Iran and 0.85 in foreign study (8, 14).

#### 3.2.2. DSM-IV Check List

DSM-IV check list was used simultaneously by two psychiatrists to agree on the same diagnosis. Its Kappa coefficient was 0.87. Clinical interview check list has been provided by Noorbala and colleagues based on DSM-IV criteria. This structural questionnaire includes 149 symptoms of mental disorders such as: symptom of mood disorders, anxiety, psychotic, psychosomatic, epilepsy, mental retardation and organic mental disorders (16). The Data were processed by SPSS software version 16 and analyzed via statistical tests include Chi -square, Kolmogrov Smirnov, Mann-whitney, independent sample t test and Leven tests.

### 3.3. Ethical Consideration

This study was approved by the Ethics Committee of the Kashan University of Medical Sciences, project number 8704. Formal consent was obtained from the prison administrator .The study protocol conformed to the ethical guidelines of the 1975 Declaration of Helsinki.

## 4. Results

In this study, 180 subjects participated in the first stage (screening). Based on SCL-90 questionnaire results, 131 subjects were referred to the psychiatrist for evaluation of mental disorders based on DSM-IV checklist. Out of 180 subjects, 21 cases were excluded because of freedom and other problems, therefore 159 subjects were interviewed by two psychiatrists. Demographic characteristic of prisoners are presented in Table 1. 

**Table 1.** Frequency of Demographic Characteristics in Male Prisoners

	Frequency	Percentage
**Age**		
15-25	40	68
26-36	68	42.7
36-50	51	32.7
**Marriage status**		
Single	65	40.8
Married	78	49.1
Divorced	16	10.1
**Occupation**		
Unemployed	9	5.7
Worker	37	23.3
Staff	7	4.4
Open	105	66.5
Retired	1	0.06
**Education**		
Illiterate	5	3.2
Primary	93	58.4
Secondary and higher	61	38.4
**History of substance **		
Yes	142	89.4
No	17	10.6
**Total**	159	100

The mean age of prisoners was 31.9 ± 8.96. Duration of imprisonment was 6.68 ± 11.82 and the frequency of imprisonment was at least 3 times. In this study between age and mental disorder there was not significant differences while, between Duration of imprisonment and mental disorder there was significant differences (Table 2). Type of crime in prisoners was selling drug (opioid substance) 67 (42.1 %), robbery and theft 47 (29.3 %), violence and disrupting social order 21 (13.2 %), and 24 (15.0%) had other crimes. Regarding to suicidal behaviors, 71 (44.6 %) prisoners had suicidal thoughts and 62 (38.9 %) history of suicidal attempt. The married prisoners had more prevalence suicidal thoughts (47.4 %) than the single prisoners. The prisoners with low level of education (elementary school) (49.5 %) had higher suicidal thought than the prisoners with high level of education.

**Table 2.** Frequency of Mental Disorder Based on Quantitative Variables (Mean age, Duration of Imprisonment)

Mental Disorder	Age, Mean ± SD	Duration of Imprisonment, Mean ± SD
**Yes**	32.26 ± 869	8.7 ± 13.79
**No**	31.65 ± 9.2	4.05 ± 7.96
**P value**	0.67	0.009

**Figure 1.**
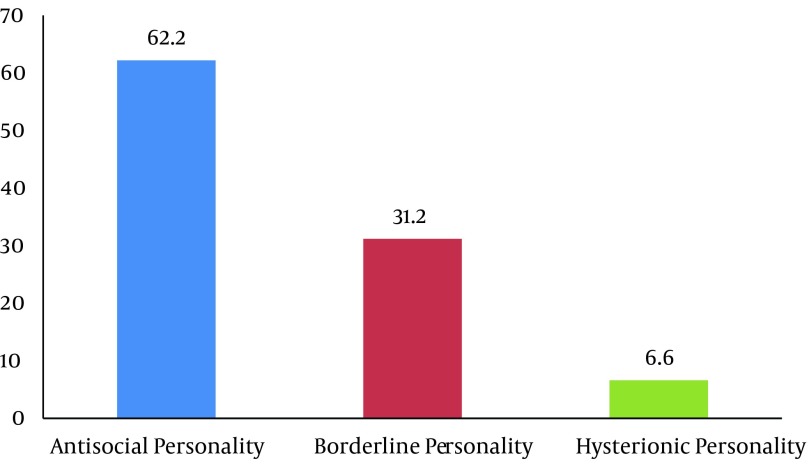
Frequency of Personality Disorders in Male Prisoners

**Figure 2.**
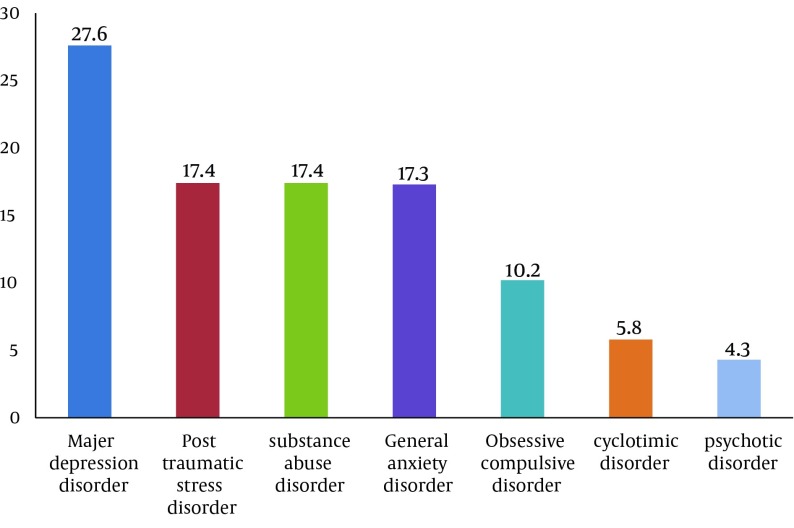
Frequency of Mental Disorders in 69 Male Prisoners Based on Clinical Interview

The Prevalence of psychiatric disorders in the prisoners was 43.4%. Mood disorders were the most prevalent psychiatric disorders. The most frequent disorder in subgroup of mood disorders was major depressive disorder (27.6 %). The prevalence of other psychiatric disorders respectively was post-traumatic stress disorder (17.4 %), substance disorder (17.4 %), general anxiety disorder (13 %), obsessive compulsive disorder (10.2 %), bipolar spectrum disorders (5.8 %) and psychotic disorder (4.3 %). Regarding comorbidity, we found high comorbidity of psychiatric disorders in the prisoners (36 %) (Excluding personality disorders). Personality disorder was found in 45 (28.3 %) based on clinical interview by two psychiatrists. Antisocial personality (62.2 %) and borderline personality disorder (31.2 %) were the most prevalent personality disorders (Figures 1 and 2).

Prisoners who had history of head trauma had more mental disorder than the others. History of head trauma in Prisoners with psychiatric disorders was 52.2 %. There was significant difference between head trauma and mental disorders (P = 0.05) (Table 3). Divorced prisoners had the most prevalent mental disorder (75 %). There was significant difference between mental disorder and marital status (P = 0.01). In this study, there was not Significant differences between mental disorder and occupational status, educational level, type of crime and history of mental disorder in the prisoners and their family (P > 0.05) (Table 2). 

**Table 3.** Frequency of Mental Disorders in Male Prisoners Based on Demographic Factors

Mental Disorder	Yes, No.(%)	No, No.(%)	Total	P value
**Marriage Status**				0.01
Single	22 (38.8)	43 (66.2)	65	
Married	35 (44.9)	43 (55.1)	78	
Divorced	12 (75)	4 (25.0 )	16	
**Mental disorders in family**				0.7
Yes	34 (44.7)	4 (55.3)	76	
No	35 (42.2)	4 (57.8)	83	
**Head trauma**				0.05
Yes	36 (52.2)	33 (47.8)	69	
No	33 (36.7)	57 (63.3)	90	
**Education**				0.3
Illiterate	2 (40.0)	3 (60.0)	5	
Primary	36 (38.7)	57 (61.3)	93	
Higher	31 (50.8)	30 (49.2)	61	
**Crime**				0.12
Robbery	15 (31.9)	32 (68.1)	47	
Violence	9 (42.9)	12 (57.1)	21	
Substance dealer	36 (53.7)	31 (46.3)	67	
Other	9 (37.5)	15 (62.5)	124	

## 5. Discussion

The present research was designed to determine the prevalence of psychiatric disorders in prisoners. In the present study, the prevalence of psychiatric disorders was 43.4 %. This prevalence is consistent with Shariat Study in Tehran prison and Abram’s study in America prison with 46. 9 %, 45. 9 % respectively (8, 17). The prevalence of mental disorders in this study are less than these studies; Tihonen and Hakula (1994) in Finland (85 %), Linda and Teplin (1997) in America (75 %), Langveld (2001) in Norway (80 %), Butler (2006) in Australia (80 %), Von Schonfeld (2006) in Germany (83.5 %), Dudeck (2009) in Germany (83 %), Brooke (1996) in England (63%), Bulten (2009) in The Neterland (57 %), Vicence (2011) in Spain (84.4%) While, in Kielsberg study (2006) the prevalence of mental disorders in Norwegian prisoners was 25 % which is less than the present study (3, 18-26).

In the present study the most prevalent mental disorders were major depressive disorder, post-traumatic stress disorder, substance disorder and general anxiety disorder which are consistent with these studies Shariat (2006) and Ashkani (2002) in Iran, Brinded (2004) in New Zealand, Abram (2003) in USA, Fazel (2002) in England, Clayer (1995) in Australia, Vergheese (1973), Medianson (1985), Bart (1998) and Hamdi (1992) (5, 7-9, 17, 27-32).

According to the results of the present study the most prevalent personality disorders were antisocial personality and borderline personality. These findings are confirmed by Modaber Arasteh (2008) in Iran, Langeveld (2001) in Norway, Von Schonfeld (2006) and Dudeck (2009) in Germany, Aghbahowe (1998) in Nijeriya, Brinded (2004) in New Zealand and Fazel (2002) in Western countries (5, 13, 19, 21, 22, 33, 34). Significant difference was not between mental disorders and age of prisoners (P = 0. 67). In Ashkani research (2002) subjects who were older than 65 years had more mental disorders that this is not compatible with present study (8). There was significant between marital status and mental disorders so that widow Prisoners had higher mental disorders compared with married and single subjects (P = 0. 01). This result is not compatible with Modaber Arasteh study (2008) in Iran (13). Jang study (2009) reported that divorced or widowed men are at high risk of depression more than other men (35).

In our study the association of mental disorders and educational level, history of substance use, family history of mental disorders and type of crime was not found (P > 0.05) (Table 2). This is in agreement with Modaber Arasteh study (2008). Suicidal ideation there was in 44. 6 % of prisoners and 38. 9 % of prisoners had history of suicidal attempts (13). Prisoners are at high risk of suicide because they do not seek help for their problems and their mental health needs were recognized less by health professional (1, 4). In addition mood and personality disorders in prisoners and other factors such as; be young, lack of social resources, substance abuse, environmental factors can be mentioned as effective factors for suicidal tendencies. This finding was confirmed by Fazel (2008), Sarchiapone (2009), Joukemaa (1997), Jenkins (2005), Lekka (2006) and Bland (1990) (34, 36-40). The present study indicates prevalence of mental disorders in prisoners with longer duration of imprisonment was higher than others. (P = 0.009). This effect can be explained by prison environment and long isolation. In evaluation type of crime and mental disorders, although substance dealers had higher mental disorders than the other offenders but this difference was not significant. This result is confirmed by Aghbahowe study (1998) (33).

This study clearly indicates that many of prisoners have mental health problems. Nearly half of prisoners who were drug dealer had mental disorder. Substance crime and mental disorder have intricate relationship and each one can cause other. This issue requires that prison services more attention to improving mental health of prisoners and prison staff and focused on the role of mental health professionals including psychiatrists, psychologists, psychiatric nurses, social workers for detection and treatment of psychiatric disorders in prison population. This study suggests that high risk prisoners are recognized and treated for prevention of consequences and vicious cycle of mental disorder and crime. In this regard prison's mental health services, should be design intervention programs in their agenda.

### 5.1. Limitations

Some limitations of our study are turnover of prisoner population, lack of cooperation of some prisoners and men samples. These limitations can reduce generalizability of this study.
